# A Clinical Audit of Pregnancy Testing in Females Presenting With Abdominal Pain to the General Surgery Department of a District General Hospital

**DOI:** 10.7759/cureus.69834

**Published:** 2024-09-21

**Authors:** Serena Patel, Niksan Thayanithy

**Affiliations:** 1 General Surgery, Imperial College NHS Trust, London, GBR; 2 Emergency Medicine, Ipswich Hospital, Ipswich, GBR

**Keywords:** quality improvement, audit, guidelines, general surgery, pregnancy testing, female, abdominal pain

## Abstract

Introduction

Guidelines state that all female patients of childbearing age presenting with acute abdominal pain to a surgical department must have a pregnancy test with either urinary or serum beta-human chorionic gonadotropin (𝜷-HCG) testing. This allows complete evaluation of the patient and consideration of a wider range of differential diagnoses, including those that must not be missed, such as a possible ectopic pregnancy. Additionally, management options for conditions unrelated to pregnancy may differ in pregnant women. This audit assessed adherence to guidelines for pregnancy testing in females presenting with abdominal pain to the general surgery department in a district general hospital and the impact of initiatives to improve compliance.

Methods

A retrospective audit to identify pregnancy test completion of all female patients aged between 11 and 55 years presenting to the general surgery department at a district general hospital with acute abdominal pain in August 2022 was conducted. A medical education session, posters, and discussion amongst multidisciplinary team members in a nursing huddle followed to raise awareness. A subsequent prospective audit was conducted in November 2022.

Results

In the initial audit conducted in August 2022, 55 female patients aged between 11 and 55 years presented to the surgical department with abdominal pain. Of these patients, pregnancy testing was only completed for 41.8% (n = 23). Following interventions, a second audit conducted in November 2022 found 30 female patients presenting with abdominal pain. In this cohort, pregnancy testing was completed for 80% of patients (n = 24).

Conclusion

This study highlights the need for regular clinical audits and multidisciplinary discussion in improving and maintaining high standards of patient care and ensuring pregnancy testing of all females of reproductive age presenting with abdominal pain to the general surgery department. Further consideration may be given to the incorporation of recording of pregnancy test status on electronic healthcare systems as part of admission and mandatory checklists.

## Introduction

An acute abdomen is defined as sudden onset intra-abdominal pathology requiring surgical intervention [1] and presents as acute onset abdominal pain. Patients with acute abdominal pain commonly attend the emergency department and account for around 10% of presentations [2]. Abdominal pain can arise due to a variety of differentials, including gastrointestinal causes such as gallstone disease, pancreatitis, and appendicitis; gynaecological pathologies such as a ruptured cyst or ectopic pregnancy; urological causes including renal colic and pyelonephritis; vascular causes such as aneurysm rupture; and medical causes including diabetic ketoacidosis and myocardial infarction. An accurate and thorough clinical assessment, including history, clinical examination, and investigations, is fundamental to safe management and correct diagnosis and treatment.

The Royal College of Surgeons of England guidelines [3] state that all female patients of childbearing age presenting with acute abdominal pain to a surgical department must have a pregnancy test with either urinary or serum beta-human chorionic gonadotropin (𝜷-HCG) testing. This allows complete evaluation of the patient and consideration of a wider range of differential diagnoses, including those that must not be missed, such as a possible ectopic pregnancy. Furthermore, management options for conditions unrelated to pregnancy may differ in pregnant women.

Additionally, the British Association of Paediatric Surgery Guidelines [4] further support this with recommendations that pregnancy testing by either urinary or serum 𝜷-HCG should be undertaken for all females aged 11 and above and girls under 11 if menses have commenced. The Royal College of Paediatricians and Child Health recognises that while over a third of females will have been sexually active by the age of 16, the likelihood of a patient aged 12-15 years being pregnant is small but tangible [5].

Notably, as well as the risks of missing a life-threatening diagnosis where pregnancy testing has not been performed, there are anaesthetic risks associated with operating under general anaesthesia. Successful outcomes after the administration of anaesthetic for non-obstetric surgery in pregnant patients are dependent upon comprehensive preoperative assessment, attention to maternal and foetal physiology perioperatively, and supportive postoperative care [6]. Studies have shown that general anaesthesia is associated with increased rates of spontaneous abortions, congenital anomalies, and low birth weights [7]. A further consideration is a risk of ionising radiation when utilising imaging modalities to reach a definitive diagnosis. Ionising radiation can be harmful to the foetus, resulting in malformations and even death depending on the radiation dose and the gestational age of the foetus, with expectant mothers needing to be adequately counselled regarding the risks if they are to require scans resulting in significant radiation doses [8,9].

This audit aimed to evaluate adherence to national guidelines for pregnancy testing of female patients of reproductive age with abdominal pain presenting to the surgical department of the district general hospital. The study also assessed interventions aimed to improve compliance, including posters and educational sessions, with a re-audit of compliance following the implementation of interventions.

## Materials and methods

This cross-sectional study was conducted in the general surgery department of a district general hospital, Ipswich Hospital, East Suffolk and North Essex NHS Foundation Trust (ESNEFT), Ipswich, Suffolk, England. It was approved by the quality improvement (QI) department of East Suffolk and North Essex NHS Foundation Trust (registration number QIP 22-362). The objective of our clinical audit was to assess whether all female patients of childbearing age presenting with acute abdominal pain to the surgical department were having pregnancy tests in the form of either urinary pregnancy (𝜷-HCG) or serum (𝜷-HCG) pregnancy testing as part of compliance with the Royal College of Surgeons England and British Association of Paediatric Surgery guidelines.

Female patients aged between 11 and 55 years presenting to the surgical department with acute abdominal pain were included. This included referrals from the emergency department of Ipswich Hospital, local general practice referrals, and other inpatient wards, and compliance referred to a documented urinary or serum pregnancy test. Patients who had previously had a hysterectomy or documented menstrual history indicating that they were post-menopausal were excluded. Data collection for the first cycle (August 1 to 14, 2022) was retrospective and included 55 patients. Data collection for the second cycle (November 16 to 30, 2022) was prospective and included 30 patients. Two reviewers meticulously examined all patients notes and blood tests to check for compliance.

The QI project used the Model for Improvement [10] approach, and the researchers were supported by a QI coach from the ESNEFT QI team.

The data collected from the initial audit underwent analysis and presentation to the local audit committee. Having identified an area for improvement, we aimed to increase the appropriate completion of urinary/serum pregnancy testing for female patients of reproductive age (defined as 11-55 years as per guidelines) presenting to surgery with acute abdominal pain.

**Figure 1:**
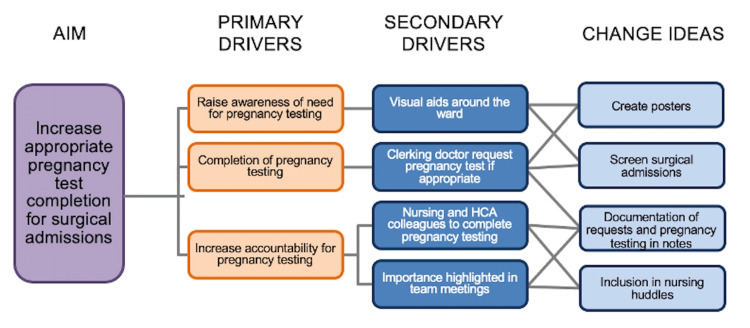
A driver diagram showing the aim of our quality improvement project and the primary and secondary drivers and change ideas identified that would support achievement of our aim. HCA: healthcare assistant

Figure 1 is a driver diagram showing drivers and change ideas to improve compliance with pregnancy testing.

Interventions were selected with the purpose of raising awareness of the need for pregnancy testing and increasing accountability for pregnancy testing amongst the surgical team. Change ideas to increase compliance included visual aids around the ward, an awareness session targeting junior doctors and nurses in the surgical assessment unit, and a discussion of the need for pregnancy testing during nursing huddles. Visual aids in the form of posters (Appendix A and Appendix B) were displayed on surgical wards, the surgical assessment unit, and the paediatric ward. 

A second dataset was collected prospectively, and the results were presented to the local audit committee showing an improvement and completion of the audit cycle.

Data analysis was conducted using Microsoft Excel (Microsoft Corp., Redmond, WA) and statistical analysis was performed using GraphPad Prism software, version 10.0.0 for Windows (GraphPad Software, Boston, MA) [9]. A chi-square test was conducted for statistical analysis, and a p-value of less than 0.05 was considered statistically significant. 

## Results

In the initial audit conducted in August 2022, 55 female patients aged between 11 and 55 years presented to the surgical department with abdominal pain. Of these patients, four were aged between 11 to 18 years, and 51 were aged between 18 and 55 years. Of these patients, pregnancy testing was only completed for 41.8% (n = 23). Of the four patients under 18 who were included, pregnancy testing was only completed in one case. Notably, one patient had a medical history of ectopic pregnancy, and pregnancy testing had not been performed in this case.

Following this first cycle, changes were implemented with the aim of improving compliance in pregnancy testing for patients presenting with abdominal pain.

A re-audit of the results was conducted in November 2022. During this period, 30 female patients aged between 11 and 55 years presented to the surgical department. Of these patients, four were aged between 11 to 18 years, and 26 were aged between 18 and 55 years. In the post-interventional audit, pregnancy testing was completed for 80% of patients (n = 24). This was a significant improvement in compliance with pregnancy testing of females of reproductive age presenting with abdominal pain to the surgical department at Ipswich Hospital (p <0.05). Pregnancy testing was completed for all of the patients under 18 who were included in this second cycle.

**Figure 2:**
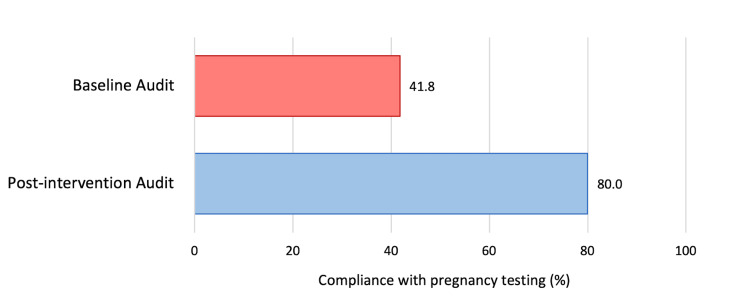
Pregnancy testing in women with abdominal pain in the baseline audit and post-intervention audit cycles In the baseline audit, pregnancy testing was completed for 23 (41.8%) patients out of 55 patients included. In the post-interventional audit, pregnancy testing was completed for 24 (80.0%) patients out of 30 patients included (p <0.05). Statistical analysis was performed using the chi-squared test.

Figure 2 shows the results of both audit cycles. 

## Discussion

Adequate and safe clinical assessment of female patients of reproductive age presenting with abdominal pain includes a prompt pregnancy test, through either urine or serum pregnancy testing. National guidelines from both the Royal College of Surgeons England [3] and the British Association of Paediatrics [5] recommend prompt pregnancy testing in these patients. This enables accurate consideration of relevant differential diagnoses and prompt safe management of patients. Undoubtedly, documentation of pregnancy status is integral to safe care, particularly if patients require general anaesthetic imaging that exposes them to ionising radiation or use of drugs that are contraindicated in pregnancy, and thus it is paramount that clinical practice reflects this.

In our study, an initial clinical audit of compliance with national guidelines at our centre showed there was room for improvement, with work needed to achieve safer levels of performance. This was evident in both pregnancy testing of adult females and of paediatric female patients presenting with abdominal pain. Similar findings were noted in a national multicentre audit of pregnancy status in general surgery admissions in Scotland [11]. This study noted that of those presenting as an emergency to general surgery, 88.1% had abdominal pain, and of these, only 65.1% had a documented pregnancy status. Furthermore, 21.3% required an emergency surgical procedure under general anaesthesia, of which 61% had a documented pregnancy status before their procedure. A further UK study [12] investigating documentation of pregnancy status, gynaecological history, date of last menstrual period, and contraceptive use in emergency surgical admissions noted only 30% of patients had a documented pregnancy status, with 32% having a documented gynaecology history, 25% having documented contraceptive use, and 29% having a documented date of last menstrual period. This highlights that our study is consistent with current practice in the UK National Health Service.

Following the results of our first cycle, medical staff received education on the importance of pregnancy testing through education sessions, inclusion in nursing huddles, and posters placed on the ward. These interventions sensitised medical staff to the need for prompt pregnancy testing and the need for documentation of this on the surgical assessment proforma. Several studies have shown that the inclusion of patient safety matters in multidisciplinary team huddles improves problem identification and improvement, situation awareness, and teamwork, resulting in better patient care [13, 14]. The effectiveness of our educational measures was assessed during our re-audit cycle, which showed a significant improvement in compliance with pregnancy testing from 41% to 80%. This highlights the pivotal role clinical audits play in improving patient care.

Whilst we did note improvement in performance in our second cycle, arguably further measures are necessary. Electronic healthcare systems should record and save pregnancy status for patients to alert clinicians when accessing records or prescribing medication to minimise errors. It may be appropriate to consider mandatory recording of pregnancy testing on electronic healthcare systems for this cohort of women on admission to the ward. Additionally, consideration should be given to the inclusion of pregnancy test status in the pre-theatre and anaesthetic checklist and also the WHO surgical safety checklist in those who are undergoing surgery under general anaesthesia, as surgical checklists have been demonstrated to be associated with increased detection of potential safety hazards and reduced surgical complications [15].

Our study has several limitations of note. Firstly, our study had a limited sample size relative to our annual general surgical admissions in both cycles of the clinical audit; a larger sample size would aid greater generalisations and more definitive conclusions to be made. Secondly, it is a single-centre study and may not be reflective of the wider experience of general surgical admissions; however, it must be acknowledged that other studies [11,12,16] including larger multi-centre studies within the NHS, have noted similar poor documentation of pregnancy status. Additionally, our study focused only on emergency presentations to the general surgery department. A further study assessing pregnancy test completion for elective surgical patients across surgical specialities could provide further valuable insight into performance and compliance with Royal College of Surgeons guidelines.

## Conclusions

This study found a significant improvement in pregnancy testing of females presenting with abdominal pain to the general surgical department following a series of interventions to improve performance after an initial audit cycle. However, further work is needed to ensure that best practice guidelines are being met and that we are providing care that is safest for our patients by accurately identifying pregnancy status as part of clinical assessment. Other measures for consideration include electronic alerts on healthcare record systems reminding clinicians of pregnancy status when accessing records or prescribing medication, and inclusion of pregnancy test status in surgical safety checklists prior to administering general anaesthesia.

## Figures and Tables

**Figure 3:**
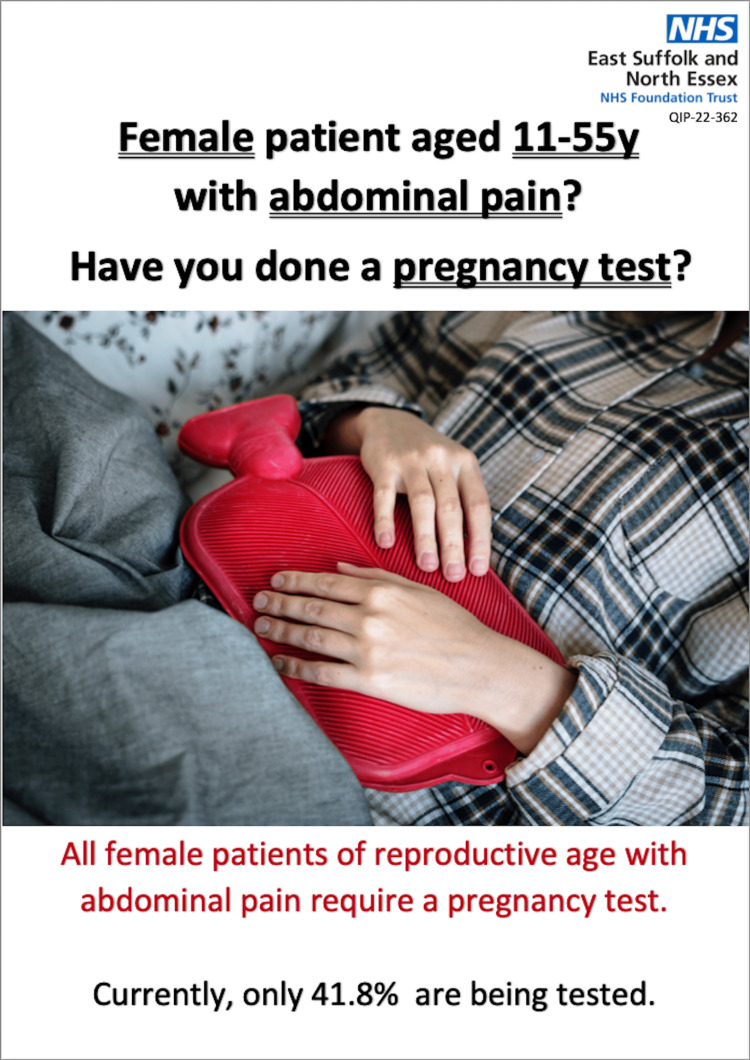
Poster displayed in the surgical wards to raise awareness of the need for pregnancy testing

**Figure 4:**
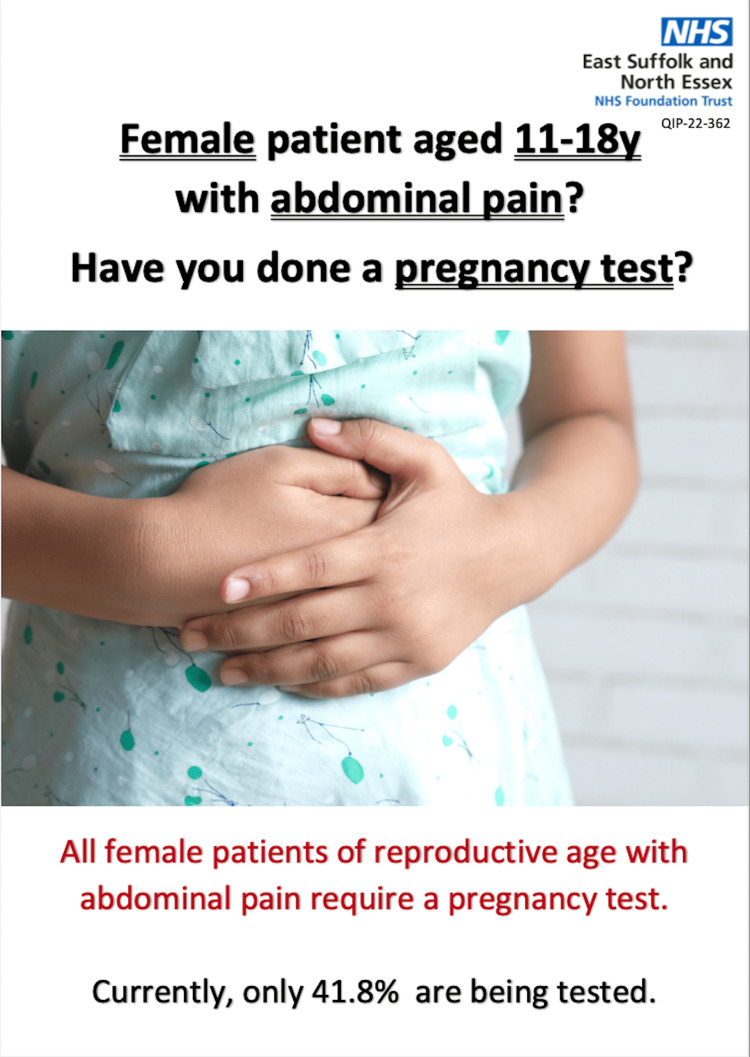
Poster displayed in the surgical wards to raise awareness of the need for pregnancy testing

## Appendices

Appendix A 

Appendix B 
